# Psychiatric neurosurgery in the 21st century: overview and the growth of deep brain stimulation

**DOI:** 10.1192/pb.bp.116.055772

**Published:** 2017-10

**Authors:** Kenneth Barrett

**Affiliations:** 1 North Staffordshire & Keele University

## Abstract

Ambulatory deep brain stimulation (DBS) became possible in the late 1980s and was initially used to treat people with movement disorders. Trials of DBS in people with treatment-resistant psychiatric disorder began in the late 1990s, initially focusing on obsessive–compulsive disorder, major depressive disorder and Tourette syndrome. Despite methodological issues, including small participant numbers and lack of consensus over brain targets, DBS is now being trialled in a wide range of psychiatric conditions. There has also been more modest increase in ablative procedures. This paper reviews these developments in the light of contemporary brain science, considers future directions and discusses why the approach has not been adopted more widely within psychiatry.

In 2014 the Mayo Clinic posted an online video in which a musician plays the violin while a neurosurgeon operates on his brain. Electrodes are being directed to an area of his thalamus with the aim of suppressing an essential tremor by electrical stimulation. Playing the violin during surgery was the best way to test whether the electrodes were in the right place. They were, and the operation was a success.^1^

Deep brain stimulation (DBS) was first used to treat severe tremor in people with Parkinson's disease in 1987 and was subsequently found effective in severe dystonia.^2^ Since then, tens of thousands of people have undergone the procedure for movement disorders and the technique has largely replaced earlier lesion-based methods. The most recent application, to ‘benign’ essential tremor, perhaps highlights its relatively low side-effect profile and patient acceptance. In 1999 a Belgian team used DBS in three people with treatment-resistant obsessive-compulsive disorder (OCD), with favourable results. There have since been DBS trials in a wide range of psychiatric conditions, and a more modest increase in ablative procedures.^3^ 2014 saw the publication of *Psychosurgery: New Techniques for Brain Disorders* by French neurosurgeon Marc Lévêque, the first ‘state of the art’ textbook to use that term in 40 years.^4^ It was a translation from the French; Anglophone practitioners in the field prefer a word with less toxic associations: neuromodulation.^5^

Whatever you call it, the use of DBS – and to a lesser extent lesion-based psychiatric neurosurgery – is on the rise, and this paper will review these developments. The aim is to inform rather than tilt opinion in a particular direction, but given the polarising nature of the subject it seems relevant to state the perspective from which it is written. The author is a retired neuropsychiatrist who formerly worked in a comprehensive service for working-age adults, within which the most frequent neurological diagnosis was acquired brain injury While in training, he obtained a doctorate in electroencephalogram-based psychophysiology. A history project on psychiatric neurosurgery in the mid-20th century led to several outcomes, including an essay that prompted the Editor to commission this review.^6^

## Background

Renewed interest in psychiatric neurosurgery has occurred against a background of major revisions in the way we think about the brain. At the beginning of the 20th century, anatomist Santiago y Cajal wrote:

‘Once brain development has ended the fount of growth and regeneration of axons and dendrites dries up irrevocably. In adult centres the nerve paths are something fixed, ended and immutable’.^7^

That ‘immutable’ view of neurons held sway within brain science for much of the past century, although there were dissenting voices, particularly between the World Wars.^8^ The brain's capacity to structurally adapt and even regenerate was eventually demonstrated at the end of the century, by magnetic resonance imaging (MRI), functional MRI (fMRI) and other techniques. In addition, simplistic notions of functional localisation are being replaced by task-related and ‘default’ systems and networks.^9^ The past 25 years have also witnessed a transformation in our understanding of glial cells. Once viewed simply as the brain's scaffolding and housekeepers, they are now known to guide brain development, shape response to injury, modulate synaptic transmission, and operate an independent chemically based communication system.^10^ How all this produces mind remains a topic rich in speculation, with recent theories encompassing ‘embodied’ cognition and quantum biology.^11,12^ The implication of all this for psychiatry remains to be determined.

In the 1930s neurologist Egas Moniz speculated that in the brains of some people with chronic mental illness, ‘the cellular bodies remain altogether normal … but their multiple liaisons, very variable in normal people … have arrangements that are more or less fixed’.^13^ He believed the most likely location for such an aberration was the pathway between the prefrontal cortex and the thalamus. In 1935/6 he tested his theory by directing a surgeon to produce small lesions in that area, in 20 patients with mental illness – the first ‘prefrontal leucotomy’ series. Moniz's targeted and theory-based approach was soon eclipsed by Freeman & Watts' more destructive and indiscriminate procedure.^14^ The extensive damage that it produced often rendered behaviourally disturbed hospital patients with psychosis docile and, in some cases, dischargeable. The potential economic benefits of that were not lost on the architects of the UK's National Health Service (NHS): in its first 5 years, there were more than 7000 leucotomies.^15^

Chlorpromazine rendered such destructive procedures in people with psychosis unnecessary and over the following 20 years psychosurgery teams – focusing on a narrower range of disorders – sought maximum benefit from minimum damage. Nonetheless, public distrust and dissenting voices increased, particularly in the USA, a trend fuelled by experiments on behaviourally disturbed children and aggressive prisoners.^16^ Beginning in 1974 a US Congressional Committee held hearings on psychosurgery, with a view to a possible ban. In the end, they recommended better regulation and, in any event, the number of operations fell dramatically over the next 30 years.^14^

Ambulatory DBS became possible in the 1980s because of technical developments (MRI, microprocessors, batteries, etc.) but also because the brain structures and pathways that produce movement had been mapped. Although the structures underlying emotion and behaviour, and the paths between them, have also been mapped, exactly how they produce the complexities of emotional life is much less clear. For that reason, a wide variety of brain areas have been targeted in this new wave of psychiatric neurosurgery. Despite the many theories as to how and why DBS and ablative procedures improve some psychiatric conditions, we do not actually know.

## Technicalities: electrodes and procedures

**Fig. 1:**
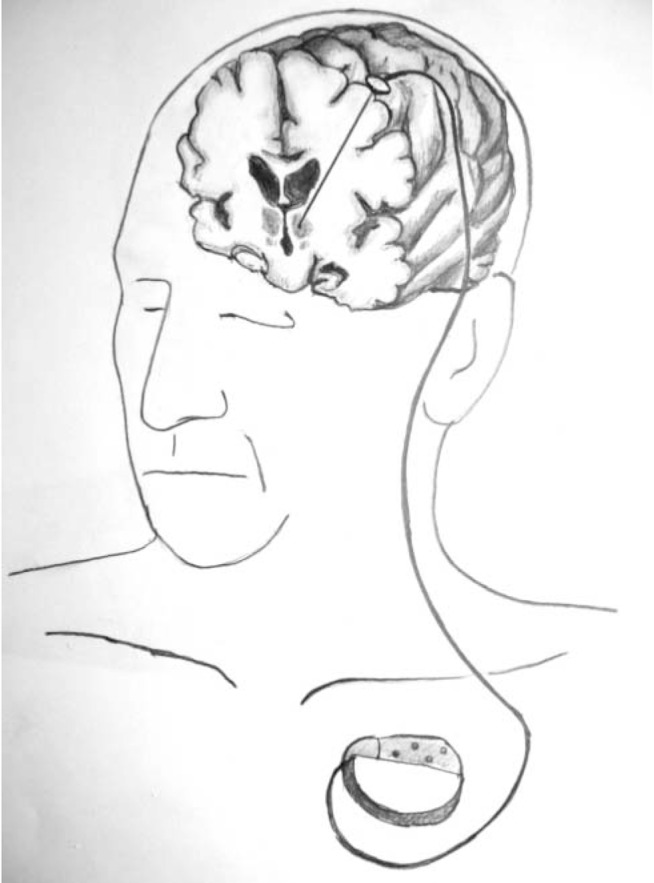
Illustration of an implanted deep brain stimulation system.

All but two of the procedures in current use involve insertion of electrodes into the brain. The exceptions are vagus nerve stimulation (VNS) and gamma knife capsulotomy. Such surgery usually involves attaching a stereotactic cage to the head as a means of directing electrodes to brain locations mapped by MRI. VNS involves isolating the left vagus nerve in the carotid sheath in the neck and looping two electrodes around it. Electrodes in both cases are wired to a programmable stimulator placed subcutaneously in the upper chest wall (Fig. 1).^17^

Each electrode includes an anode and a cathode. When an electrical current is applied the brain tissue between them joins the circuit. Three to five volts are usually applied in DBS at pulse frequencies above 100/s. At such frequencies brain tissue immediately surrounding the electrodes is deactivated/depolarised. However, just outside that area, volume conduction leads to electrical stimulation of axons, propagated upstream to cell bodies and downstream to synapses, interrupting local brain function while also producing effects more remotely. At frequencies below 100 – 15 pulses/s, for example, in VNS – stimulation is also produced in the tissue immediately surrounding the electrodes. Electrodes have been used experimentally to stimulate brain tissue in humans since the late 1940s.^18^ They have also been used to produce lesions, through thermocoagulation. In anterior cingulotomy, for example, a 10 mm exposed portion of the electrode is heated to 85°C for 60 s.

In gamma knife surgery, multiple narrow beams of gamma radiation intersect at a pre-mapped point in the brain, hence the skull is not opened.^17^

## Brain targets and treatment rationales

Box 1 summarises the brain targets used in most psychiatric neurosurgery over the past 20 years, and the conditions treated in each case. The targets for current lesion surgery are those that have been found to produce the most benefit with the fewest adverse effects. The targets used for psychiatric DBS were chosen in four ways.

1. The first trial, in 1999, targeted the anterior limb of the internal capsule because lesion surgery to that area has been found in some cases to reduce the symptoms of severe OCD.^3^ The anterior cingulate and subcaudate areas, and the combination of the two, were chosen for similar reasons, in relation to major depressive disorder (MDD).
2. The subthalamic nucleus (STN) is the favoured target in Parkinson's disease. Following DBS, some patients with comorbid OCD experienced a reduction in the severity of those symptoms, hence its selection for trials in OCD.
3. fMRI has revealed increased metabolic activity in the subgenual cortex and habenula in some patients with MDD. Hence, those areas were targeted based on the hypothesis that such hyperactivity may be causal, rather than simply a manifestation of depression.
4. Tourette syndrome sits on the boundary between movement and compulsive disorder, which has contributed to the wide range of brain targets available, including the thalamus, STN, globus pallidum, nucleus accumbens and internal capsule.

**Box 1** Brain targets used in psychiatric neurosurgery

**Stimulation procedures**

**Deep brain**

- Anterior limb internal capsule (obsessive-compulsive disorder (OCD), depression, anorexia nervosa)
- Nucleus accumbens (OCD, depression, anorexia nervosa, addictions)
- Subgenual cortex (depression)
- Globus pallidus (Tourette syndrome)
- Habenula (depression)
- Posterior hypothalamus (aggressive behaviour)
- Thalamus centromedian nucleus (Tourette syndrome)
- Subthalamic nucleus (OCD)
- Inferior thalamic peduncle (depression)
- Nucleus basalis (Alzheimer's disease)
- Fornix (Alzheimer's disease)
- Basolateral amygdala (post-traumatic stress disorder)

**Cortical surface (epidural)**

- Dorsolateral frontal (depression)
- Orbitofrontal (depression)

**Vagus nerve (depression)**

**Ablative procedures**

**Thermocoagulation**

- Anterior capsulotomy (OCD, depression)
- Cingulotomy (OCD, depression, addiction)
- Limbic leucotomy (OCD)
- Subcaudate tractotomy (depression, OCD)
- Nucleus accumbens (addiction)

**Radiosurgery (‘gamma knife’)**

- Capsulotomy (OCD)

VNS was first used in the 1990s to treat some forms of refractory epilepsy. Improvement in the mood of some individuals was noticed, an effect that was independent of seizure response. Trials in treatment-resistant depression yielded positive outcomes, but a later study including a ‘sham’ surgery group suggested a significant placebo effect.^19^

Several authors have sought to explain the beneficial effects of stimulation-based procedures and lesion surgery on depressed mood and anxiety by reference to two cortico-striato-thalamocortical (CSTQ ‘loops’.^20^ Similar loops were previously identified in relation to movement, prior to the introduction of DBS, including inhibitory (GABA-based) and excitatory (glutamate-based) pathways.^21^ The CSTC loops, by contrast, involve a wider range of neurotransmitters, with complex interactions that are yet to be defined. Such ‘circuits’ may, however, explain the variety of targets that seem to produce at least some benefit in psychiatric DBS studies. It seems reasonable to speculate that tapping into and stimulating the loop at many points could influence and modify the whole network.

## Protocols, conditions and outcomes

Lesion-based, ablative surgery continues to be available, including at two centres in the UK, but in most countries it is confined to a very small number of cases each year.^22,23^ The exceptions are Russia and China where it is now frequently used in the treatment of addiction.^24,25^ Radiosurgery has made lesion surgery possible without opening the skull and one report confirmed efficacy in OCD comparable to older techniques.^26^ At the Editor's direction, the remainder of this review will focus on DBS.

The investigators who first applied DBS to the treatment of psychiatric disorders were aware of the legacy of past psychosurgical excesses and the ethical issues it raised. With that in mind a collaborative group drew up a list of research guidelines, published in 2002.^27^ They include independent evaluation of potential participants according to strict diagnostic, severity and duration criteria; the need to ensure that individuals are able to give informed consent at the outset and for as long as the treatment continues; and ensuring DBS is never used for ‘political, law enforcement or social purposes’.^27^ Most investigators also use established severity ratings and response criteria based on them (such as a 35% or more reduction on the Yale-Brown Obsessive Compulsive Scale^28^).

In the early years the focus of DBS studies was on people with treatment-resistant OCD, Tourette syndrome and MDD. The textbook cited at the beginning of this paper tabulates all such studies up to 2013 for each of these diagnoses, including patient numbers, brain targets, follow-up times and reported outcomes.^29^ In summary:

- OCD: 11 studies involving 9 targets in a total of 86 patients followed up from 3 to 31 months; positive outcomes in 33–100%
- Tourette syndrome: 10 studies involving 7 targets in 40 patients followed up from 3 to 36 months; positive outcomes in 23–82%
- MDD: 6 studies involving 5 targets in 55 patients followed up from 12 to 36 months; positive outcomes in 30–75%.

The longer the duration of the follow-up overall, the better outcomes tended to be, but no one target appeared markedly superior. Whereas in movement disorder, and to an extent in Tourette syndrome, improvement occurred soon after stimulation commenced, in OCD and MDD improvement took many weeks to begin, symptoms diminishing further as time progressed. This perhaps indicates that rather than simply turning off ‘malfunctioning’ neurons, stimulation causes gradual beneficial change in the networks and systems it taps into. One exception to this delayed response was a study in which seven people with MDD received DBS to the medial forebrain bundle. The pulse frequency was lower than usual (see ‘Technicalities’ section) and at 1 year positive responses were reported in six people; all began to improve within a week of stimulation commencing.^30^

Despite these apparently favourable outcomes, the small patient numbers, bewildering array of brain targets, variable follow-up times, and the impossibility of the double-blind placebo control methods, may lead many to conclude that the efficacy of this approach is far from proven. Although Lévêque considers the efficacy of DBS in OCD to be established, in relation to MDD he concedes that ‘although the benign nature of these techniques is in the process of being established, their efficacy remains difficult to demonstrate’.^29^ Nonetheless, he and others offer several reasons why these outcomes should be taken seriously.

1. The patients treated have severe conditions that have failed to respond to all other treatments over a prolonged period.
2. Some studies target structures that had proved effective in lesion-based surgery (stimulation being used to simulate a lesion).
3. DBS allows for a form of ‘double blind’ methodology as the stimulating device may be turned on and off, the status at any point being kept from patient and assessor.
4. In some clinically improved cases temporary deterioration followed battery failure or inadvertent disconnection.^30,31^

Although the reversible nature of DBS makes it more acceptable than lesion surgery, is it as effective in psychiatric applications, and does it result in fewer side-effects? The technique may only simulate a lesion, but if the patient requires that simulation to be in place for the rest of their life, what is the difference, apart from indefinite maintenance costs? A recent paper addressed that question by reviewing outcomes in 20 studies of treatment-resistant OCD.^32^ 108 patients who underwent capsulotomy were compared with 62 patients who received stimulation to the internal capsule or the nucleus accumbens. Of those undergoing capsulotomy 62% responded favourably, compared with 52% of those undergoing stimulation, but the difference was not statistically significant. Weight gain, which was common after lesion surgery, did not occur with stimulation. Apathy and disinhibition were also experienced by a small number of patients after lesion surgery but not during DBS.

Adverse effects reported after DBS include postoperative problems such as wound infection, haemorrhage (asymptomatic or resulting in transient motor signs), single seizures and syncopal episodes.^33^ Additional undesirable effects develop when the stimulator is turned on but seem generally to disappear once the stimulation parameters are altered (voltage, frequency, etc.). These include physical symptoms such as paraesthesia, muscle contractions, dysarthria, diplopia and strabismus, and psychiatric features, particularly excitement, irritability and occasionally hypomania. Cognitive function is usually assessed before and during treatment and a recent review concluded that no adverse cognitive effects had occurred. In fact, as time progressed improvements in scores tended to occur, mirroring improvements in mental state.^34^ The most common longer-term problems in psychiatric and movement disorder applications seem to be device-based. For example, one study of 84 patients with Parkinson's disease recorded hardware-related complications in 8.4% of patients each year, including lead fractures, migrations and disconnections.^35^ Technical improvements have doubtless occurred since that report, but the consequences of such mishaps in patients with severe psychiatric disorders may be grave, and are among the reasons that regular ongoing follow-up is deemed important.

A further stimulation technique that does not involve penetrating the brain has recently been tried in people with MDD.^36^ Electrodes were placed in the epidural space over the dorsolateral frontal cortex in a single-blind study of 12 patients who were followed up for 2 years, with results comparable to the best DBS studies. A later paper discussed the combination of this technique with psychotherapy, a fascinating subject, sadly beyond the scope of this review.^37^

The major advantage of DBS over lesion-based surgery is that if it does not work the hardware can be turned off and removed. In the longer term, stimulation could be stopped temporarily to assess whether it is still necessary. But as we now know, the brain is not ‘immutable’ and unresponsive to such challenge. It adjusts and adapts, chemically and structurally to changing circumstances and, in fact, the delayed onset of improvement in some of these applications seems to depend on such adaptation. The long-term implications of such changes are not clear.

In light of the relatively low side-effect profile of DBS in these early trials and apparently favourable outcomes, the technique has been extended to a number of other conditions including addiction,^38^ eating disorder,^39^ posttraumatic stress disorder (PTSD),^40^ early Alzheimer's disease^41^ and, most controversially, aggressive behaviour disorder.^42^ Each of these has a defining clinical feature that suggests a particular brain target (in turn, nucleus accumbens, hypothalamus, amygdala, mammillary-fornix-hippocampal complex/nucleus basalis, hypothalamus). Although it is probably too early to comment on the outcome of this work, the target selections in two of these conditions are illustrative of the current approach and will be described briefly. The amygdala is being targeted in PTSD as a result of post brain-injury MRI and fMRI evidence (amygdala damage protecting against developing the condition and evidence of increased metabolic activity), and a positive response to amygdala stimulation in an animal model. The fornix is being targeted in early Alzheimer's disease as a way into the mammillary-fornix-hippocampal complex. This follows the serendipitous finding of improved memory and increased hippocampal volume following stimulation of the anterior hypothalamus undertaken for an unrelated condition, and animal studies showing stimulation-related neural growth.^43^

## The future

In 2013 President Obama launched a US$100 million research programme with the acronym B.R.A.I.N (Brain Research through Advancing Innovative Neurotechnologies).^44^ ‘Emerging technologies’ would be applied to the investigation of brain function and the treatment of disorders. This would include nanotechnology and, in relation to treatments, ‘wireless fully implantable neural interface medical devices for human use … closed loop systems able to deliver targeted neural stimulation’.^45^ A patient group singled out for such innovative treatments was injured war-fighters, particularly those with treatment-resistant PTSD and memory problems due to acquired brain injury. This was a remarkable proposal, not least because at that point no ‘wired’ device-based treatments had been found useful or even trialled in either condition, and closed loop technology had only been used in cardiac dysrhythmia and epilepsy.^46^

Closed loop technology has been more widely identified as important for the future of this work. In the context of epilepsy, implanted closed loop devices monitor an area where seizure originates, detect electrical activity that indicate a seizure is due, and respond with electrical stimulation or cooling to interrupt it.^46^ In the psychiatric context, the possibilities of such devices include using nanotechnology to measure neurotransmitter levels and trigger therapeutic outputs.^47^

Another development of possible importance is optogenetics. Light-sensitive ion channels that respond to different colours are delivered to and incorporated into individual neurons via a virus. Light channelled into the brain via fibroptics can then be used to turn on and off such channels. Although this sounds the stuff of science fiction, a similar procedure has apparently succeeded in animal studies and trials in humans are expected in due course.^48,49^

## Conclusions

Given the torment of severe treatment-resistant depression, OCD and other psychiatric conditions, and the enthusiasm for DBS in relation to movement disorder, it is reasonable to ask why the technique has not been more widely adopted in psychiatry? A neurosurgeon recently addressed this question and identified a number of reasons: the legacy of ‘old-fashioned’ psychiatric surgery, the complexity and heterogeneity of psychiatric symptoms, and the multitude of brain circuits likely to be involved in them, ‘tricky ethical questions related to potential manipulation of the mind’, difficulty in conducting large trials in these conditions, and inconsistent results.^50^ He might have added lack of consensus over targets in the conditions most often treated and the limited number of ‘placebo’ (sham treatment) controlled trials. Nonetheless, the US Food and Drug Administration gave approval for DBS in treatment-resistant OCD in 2009, albeit through a ‘humanitarian device exemption’.^33^

In the UK, new medical procedures tend to be adopted and funded following pressure from patients and their interest groups, clinicians, medical Royal Colleges and the media. It is difficult to envisage such pressure for psychiatric DBS at the moment. The legacy of mid-20th century psychosurgery includes not only public and professional distrust (the charity OCD-UK ‘do not recommend DBS as a treatment for OCD *and remain concerned that the dangers associated with the procedure continue to be overlooked by the medical community’*^51^ – my italics) but also ethical and methodological rules that require complex, expensive, multiprofessional teams. In 2013 the NHS Commissioning Board published DBS guidelines for the treatment of movement disorders. They included an estimated cost of £26 070 for each procedure, but acknowledge that savings resulting from clinical and quality-of-life improvements offset ongoing maintenance costs.^52^

In an afterword to the psychosurgery text mentioned at the beginning of this paper, and a related article, Marwan Hariz, a particularly cautious and thoughtful ‘functional’ neurosurgeon, warns that ‘hyping’ DBS in psychiatry at this stage could lead to its demise.^50^ He also expresses concern at recent suggestions that the technique could be used to enhance ‘normal’ functioning or even control antisocial behaviour. ‘Neuromodulation’ he concludes, ‘should not be allowed to become neuromanipulation’.^53^ It remains to be seen whether a new generation of ‘millennial’ psychiatrists and neurosurgeons armed with these emerging technologies will follow his advice.
